# A Practical Treatise on Mechanical Dentistry

**Published:** 1893-12

**Authors:** 


### Bibliography.

A Practical Treatise on Mechanical Dentistry. By Joseph Richardson, M.D., D.D.S. Sixth Edition. Revised and edited by George W. Warren, D.D.S. With six hundred illustrations. P. Blakiston, Son & Co. Philadelphia, 1893.

The appearance of the sixth edition of this well-known work on mechanical dentistry will be received with gratification by the dental profession. It has for so long a period been a standard book on this subject that any lengthy review would seem unnecessary.

It is a satisfaction to find that the original work, so ably prepared by the lamented author, Dr. Richardson, has been carefully and thoroughly supplemented by the labor of Dr. Warren.

The task of preparing this edition must have been very heavy; indeed, it may be judged nearly equal to the original preparation. It is a pleasure to notice this careful revision, as it leads to the hope that future editions of other works published by this firm may receive similar treatment. There has been entirely too much of a repetition of old and obsolete matter from edition to edition, until publications have ceased to have any value either as text-books or works of reference.

Crown- and bridge-work has been so rapidly developed in recent years that a new edition required that this part should be almost entirely rewritten, and this has been accomplished in 202 pages,—

almost a book of itself. This work of the editor is in the main complete and satisfactory in the general processes given, but we look in vain for some regarded by experts as of special value, notably the movable bridge of Dr. Richmond; indeed the omission of the work of this excellent mechanician seems a positive mistake, and should be corrected in future editions. There is no part of a book that should be more carefully guarded than that of giving credit for original work.

The illustrations are profuse and generally excellent, but some from an artistic point of view are not worthy the pages upon which they are printed, the teeth in several instances resembling nothing in nature. Aside from these defects this part of the book is very well done, and exhibits great care to bring this difficult subject up to date, and at the same time make it clear to the reader.

While there may be a difference of opinion as to where the line should be drawn between operative and mechanical dentistry, it appears that certain processes usually claimed for the former belong, in part at least, to the latter. In this class may be placed the preparation of regulating plates. While these vary constantly to meet changing conditions, it is clear that the description of certain fixed forms would be of great advantage to the student. This has been entirely omitted in this edition.

The general make-up deserves the highest commendation, and corresponds in this respect with the very careful work of this publishing house.

This volume in the present as in the past preserves its reputation of being the only work on mechanical dentistry worthy to be heartily recommended as a text-book for students and for continual reference by those in practice.

